# Effects of He and Ar Heat-Assisted Plasma Treatments on the Adhesion Properties of Polytetrafluoroethylene (PTFE)

**DOI:** 10.3390/polym13234266

**Published:** 2021-12-06

**Authors:** Yuji Ohkubo, Yuki Okazaki, Masafumi Shibahara, Misa Nishino, Yosuke Seto, Katsuyoshi Endo, Kazuya Yamamura

**Affiliations:** 1 Graduate School of Engineering, Osaka University, 2-1 Yamadaoka, Suita 565-0871, Japan; y-okazaki@div1.upst.eng.osaka-u.ac.jp (Y.O.); m-nishino@div1.upst.eng.osaka-u.ac.jp (M.N.); y-seto@div1.upst.eng.osaka-u.ac.jp (Y.S.); endo@upst.eng.osaka-u.ac.jp (K.E.); yamamura@prec.eng.osaka-u.ac.jp (K.Y.); 2 Materials and Analysis Department, Hyogo Prefectural Institute of Technology, 3-1-12 Yukihiracho, Kobe 654-0037, Japan; shibahara@hyogo-kg.jp

**Keywords:** adhesion, plasma gaseous species, surface treatment depth (surface modification depth), fluoropolymers, heat-assisted plasma treatment

## Abstract

Heat-assisted plasma (HAP) treatment using He gas is known to improve the adhesive-bonding and adhesive-free adhesion properties of polytetrafluoroethylene (PTFE). In this study, we investigated the effects of He and Ar gaseous species on the HAP-treated PTFE surface. Epoxy (EP) adhesive-coated stainless steel (SUS304) and isobutylene–isoprene rubber (IIR) were used as adherents for the evaluation of the adhesive-bonding and adhesive-free adhesion properties of PTFE. In the case of adhesive bonding, the PTFE/EP-adhesive/SUS304 adhesion strength of the Ar-HAP-treated PTFE was the same as that of the He-HAP-treated PTFE. In the case of adhesive-free adhesion, the PTFE/IIR adhesion strength of the Ar-HAP-treated PTFE was seven times lower than that of the He-HAP-treated PTFE. The relation among gaseous species used in HAP treatment, adhesion properties, peroxy radical density ratio, surface chemical composition, surface modification depth, surface morphology, surface hardness, and the effect of irradiation with vacuum ultraviolet (VUV) and UV photons were investigated. The different adhesive-free adhesion properties obtained by the two treatments resulted from the changes in surface chemical composition, especially the ratios of oxygen-containing functional groups and C–C crosslinks.

## 1. Introduction

Polytetrafluoroethylene (PTFE) is a typical fluoropolymer consisting of only CF_2_ chains. It exhibits high sliding properties, high hydrophobic and oleophobic properties, high electric insulation, high ultraviolet (UV) resistance, and high heat resistance. So, it is applicable under severe environments even though other polymers are unavailable for the situation because of a shortage of some properties. However, it has extremely low adhesion strength, low mechanical durability, and high cost. Therefore, the multi-materialization of PTFE is vitally important. Surface modification is required for PTFE because of its low adhesion properties. UV irradiation [1,2,3], electron beam (EB) irradiation [4,5], and synchrotron irradiation [6] have been used to improve the adhesion properties of PTFE; however, these methods concern a deep modification depth and thus were excluded from the present study. Although various surface modification methods for polymers such as corrosive solution immersion treatment [7], plasma treatment [8,9,10,11], and ion irradiation [12,13,14] have been developed, the most effective methods for PTFE modification include Na-containing solution immersion treatment (Na treatment) [15,16,17], surface graft polymerization after plasma treatment [18,19,20], surface graft polymerization during plasma treatment [21,22,23], and heat-assisted plasma (HAP) treatment [24]. Conventional plasma treatments have given a low effect of improvement in the adhesion property for PTFE [8,9,10,11]; however, Ohkubo et al. found that heating during plasma treatment positively affected the adhesion property of PTFE. Although the HAP treatment enables PTFE to strongly adhere to several materials, such as epoxy (EP)-based adhesive, isobutylene–isoprene rubber (IIR), and polydimethylsiloxane (PDMS), as a plasma process gas, specific problems are that He gas is very expensive and more unstably supplied than other gases, such as Ar and N_2_. Although plasma gaseous species such as O_2_, CF_4_, Ar, air, He, Ar + N_2_ + C_2_H_2_, and Ar + NH_3_ + H_2_O have been compared [25], the effects of plasma gaseous species on HAP treatment have not been not investigated. Ar is an inert and safe gas as well as He, and in addition, Ar gas is less expensive and more stably supplied than He gas. In this study, we compared the effects of Ar-HAP treatment with those of He-HAP treatment on the adhesion property, peroxy radical density, surface chemical composition, surface modification depth, surface morphology, and the surface hardness of PTFE.

## 2. Materials and Methods

### 2.1. Materials

Rolled PTFE sheets (300 mm × 10 m × *t*0.2 mm, NITOFLON^®^ No.900UL) were purchased from Nitto Denko (Kita-ku, Osaka, Japan). The rolled PTFE sheets were cut into dimensions of 70 mm × 45 mm × *t*0.2 mm. Prior to HAP treatment, the PTFE sheets were washed with acetone (99.5%, Kishida Chemical, Chuo-ku, Osaka, Japan) and pure water using an ultrasonic bath (US-4R, AS-ONE, Nishi-ku, Osaka, Japan) for 1 min, respectively. Then, the washed PTFE sheets were dried using a spray gun with N_2_ gas (99.99%, Iwatani Fine Gas, Amagasaki, Hyogo, Japan). Two types of adherents, stainless steel (SUS304) flat bar coated with EP-based adhesive and unvulcanized IIR, were used in the present study, because obtained results often differ between adhesive bonding using an adhesive and adhesive-free adhesion without any adhesive [26]. In adhesive bonding, SUS304 flat bars (10 mm × 175 mm × *t*3 mm, MISUMI, Bunkyo-ku, Tokyo, Japan) and a two-component EP adhesive (epoxy resin AV-138 and hardener HV-998, Nagase ChemteX, Nishi-ku, Osaka, Japan) were purchased and used. The SUS304 flat bars were washed and wiped with acetone (99.5%, Kishida Chemical, Chuo-ku, Osaka, Japan) before use. In the adhesive-free adhesion, unvulcanized IIR sheets with the ca. 2-mm thickness were prepared based on a previous patent [27]. The IIR sheets were not washed before use.

### 2.2. Methods

**Figure 1:**
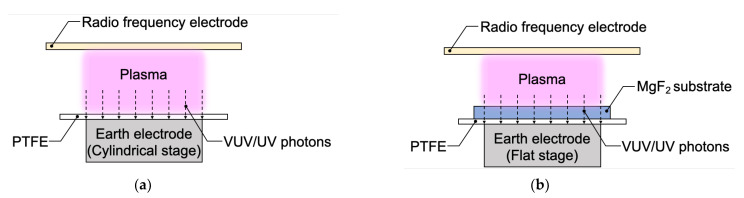
Schematic depicting the irradiation with vacuum ultraviolet (VUV) and UV photons to the PTFE surface during plasma treatment (**a**) without using an MgF_2_ substrate and (**b**) using an MgF_2_ substrate.

In this study, the influences of gaseous species used in HAP treatment on the adhesion properties of PTFE were investigated. According to our previous reports, He gas (99.99%, Iwatani Fine Gas, Amagasaki, Hyogo, Japan) was used as a process gas. In comparison to He gas, Ar gas (99.99%, Iwatani Fine Gas, Amagasaki, Hyogo, Japan) was also used. Except for gaseous species and applied radio-frequency (RF) power density, HAP treatment was performed as previously reported [24]. Firstly, PTFE sheets were fixed on the cylindrical rotation stage of the earth electrode, as shown in Figure 1a. Secondly, the pressure in a custom-made plasma reactor (Meisyo Kiko, Tanba, Hyogo, Japan) was decreased from atmospheric pressure (101,300 Pa) to <10 Pa using a rotary vacuum pump (EC603, Ulvac, Chigasaki, Kanagawa, Japan). Thirdly, a process gas (He or Ar) was introduced from <10 Pa to atmospheric pressure (101,300 Pa). Fourthly, He or Ar plasma was generated between two electrodes. Applied RF power density for either He or Ar plasma generation was set to 5.9 W/cm^2^ because Ar plasma was significantly unstable when a relatively high RF power density, such as 10 W/cm^2^, was applied. Finally, PTFE sheets were plasma-treated for 600 s. A near-infrared-radiation line heater (KSC100-24/OU, K-Sonic, Koshigaya, Saitama, Japan) was used to heat the PTFE surface during plasma treatment, and the line heater was cooled using a low-temperature circulator (LTC-1200N, AS-ONE, Nishi-ku, Osaka, Japan). The surface temperature of PTFE was monitored using a digital radiation thermometer system (FT-H40K and FT-50A, Keyence, Higashiyodogawa-ku, Osaka, Japan) and maintained at 200 °C in HAP treatment. In addition, the effect of irradiation with vacuum ultraviolet (VUV) and UV photons during plasma treatment was investigated using an MgF_2_ substrate (40.0 mm × 20.0 mm × *t*0.1 mm, M40-20-0.1, Pier Optics, Tatebayashi, Gunma, Japan) to separate the effects of electrons, ions, and metastable excitons and the VUV/UV irradiation, as reported previously [25,28]. In summary, an MgF_2_ substrate was fixed on the surface of PTFE samples using a flat stage of the earth electrode to protect the PTFE surface from contact with electrons, ions, and metastable excitons in plasma; then, the PTFE surface was irradiated with VUV/UV photons through the MgF_2_ substrate, as shown in Figure 1b.

### 2.3. Analysis

#### 2.3.1. Evaluation of Adhesion Properties

For the evaluation of the adhesion properties of PTFE in the presence of an adhesive, PTFE/EP-adhesive/SUS304 assemblies with a width of ca. 10 mm were prepared as reported previously [26]. HAP-treated PTFE samples were adhered to a SUS304 flat bar using a two-component EP adhesive (epoxy resin AV-138 and hardener HV-998, Nagase ChemteX, Nishi-ku, Osaka, Japan). The EP adhesive was cured at 80 °C for 30 min using a hot plate (HHP-170D, AS-ONE, Nishi-ku, Osaka, Japan). To measure the adhesion strengths of the PTFE/EP-adhesive/SUS304 assemblies, 90° peel tests were performed using a digital force gauge (ZP-200N, Imada, Toyohashi, Aichi, Japan) and an electrically driven stand (MX-500N, Imada, Toyohashi, Aichi, Japan). For the evaluation of the adhesive-free adhesion properties of PTFE, PTFE/IIR assemblies with a width of ca. 10 mm were prepared [26]. HAP-treated PTFE samples were adhered to an unvulcanized IIR sheet in a mold. The PTFE/IIR assemblies were vulcanized and adhered simultaneously at 180 °C at 10 MPa for 10 min using a hot-pressing machine (AH-2003, AS-ONE, Nishi-ku, Osaka, Japan). To measure the adhesion strengths of the PTFE/IIR assemblies, T-peel tests were performed using the same digital force gauge and electrically driven stand as the 90° peel tests. HAP treatments were performed in triplicate for each plasma condition, and the three adhesion strengths were averaged.

#### 2.3.2. Evaluation of Peroxy Radicals

To confirm the generation of a peroxy radical (C–O–O^•^) upon plasma treatment and compare the radical density ratios of plasma-treated PTFE samples, electron spin resonance (ESR) measurements were performed at room temperature using an ESR spectrometer (JES-FA100x, JEOL, Akishima, Tokyo, Japan) with an X band. The plasma-treated PTFE samples with dimensions of 3 mm × 30 mm × *t*0.2 mm were inserted into a quartz glass cell. The microwave power was 10 mW, and the applied frequency was 10 GHz. ESR spectra were recorded at 328.5–343.5 mT. Mn^2+^ in MgO containing Mn ions was used as a reference. The ESR spectra of the as-received and the plasma-treated PTFE samples are shown in Figures S1 and S2 (Supplementary Material). Except for the ESR spectra of the as-received PTFE, broad peaks indexed to the peroxy radical were observed between 332 and 337 mT in all ESR spectra. Radical density ratios were calculated by the double integration of the signal intensity indexed to peroxy radicals in the ESR spectra using software (JFS-FA cwESR Ver. 3.0.0.1, JEOL, Akishima, Tokyo, Japan).

#### 2.3.3. Evaluation of Surface Chemical Composition

To confirm the generation of oxygen-containing functional groups (C=O–O, C=O, C–O) and C–C crosslinks upon plasma treatment and compare the ratios of the functional groups on the plasma-treated PTFE samples, X-ray photoelectron spectroscopy (XPS) measurements were performed using an XPS spectrometer (Quantum 2000, Ulvac-Phi, Chigasaki, Kanagawa, Japan). The procedure of XPS measurements was the same as previously reported [24]. Narrow scan XPS spectra of C1s and O1s were obtained at 295–283 and 540–525 eV, respectively. The O1s-XPS spectra of the PTFE samples HAP-treated using He or Ar gas with heating are shown in Figure S3. The step size was 0.05 eV, and the accumulated measurements were performed three times. To prevent the PTFE samples from charging, a low-speed EB and an Ar ion beam were irradiated to the PTFE samples during XPS measurements. The obtained XPS spectra of plasma-treated PTFE samples were referenced to peaks indexed to –CF_2_– at 291.8 eV [29,30], while only the obtained XPS spectrum of the as-received PTFE sample was referenced to peaks indexed to –CF_2_– at 292.5 eV [31,32]. All the peak intensities were normalized based on the intensity of the peak indexed to CF_2_ for the as-received PTFE sample. Atomic ratios were calculated using data analysis software (MultiPak Ver. 8.2C, Ulvac-Phi, Chigasaki, Kanagawa, Japan). Peak resolution of C1s-XPS spectra was performed, and the functional-group ratios were calculated using data analysis software (XPSPEAK Ver. 4.1, Free download). The background type was “Shirley”.

#### 2.3.4. Measurement of Plasma Surface Modification Depth

To investigate the depth of surface modification on the HAP-treated PTFE samples, a combination of XPS measurements and soft etching using a gas cluster ion beam (GCIB) were alternately performed using an XPS spectrometer (PHI5000Versa ProbeII, Ulvac-Phi, Chigasaki, Kanagawa, Japan), which is known as a GCIB–XPS measurement. An Ar^+^ GCIB was irradiated at 5 kV to the PTFE samples with an irradiation area of 2 mm × 2 mm. One cycle time of the GCIB soft etching was 0.5 min, and the number of the total cycles was 30. Before the GCIB–XPS measurement, the sputtering rate of the GCIB for the as-received PTFE was calculated by dividing the etched depth by the etching time. The GCIB sputtering hall was observed using a scanning white-light interferometer (SWLI, NewView8000, Zygo, Middlefield, CT, USA), as shown in Figure S4. When the time of the GCIB soft etching was 2000 min, the GCIB etching depth was 7 ± 1 μm; thus, the calculated sputtering rate was 3.5 ± 0.5 nm/min. The GCIB–XPS measurements were performed until no oxygen-containing functional groups could be detected. Finally, the depth of surface modification was calculated by multiplying the calculated sputtering rate (3.5 ± 0.5 nm/min) by the total number of GCIB sputtering needed for no detection of oxygen-containing functional groups.

#### 2.3.5. Confirmation of Removal of Weak Boundary Layer (WBL)

The existence of a weak boundary layer (WBL) remarkably decreases the adhesion property of PTFE. Therefore, the confirmation of the presence or absence of a WBL on PTFE is extremely important. To investigate the change in surface morphology via plasma treatment, especially to confirm whether a WBL on the as-received PTFE was removed and recovered via HAP treatment or not, the plasma-treated PTFE samples were observed using a scanning electron microscope (SEM, JCM-6000, JEOL, Akishima, Tokyo, Japan). Before SEM observation, the HAP-treated PTFE samples were covered with an ultrathin Au film using an ion sputtering apparatus (Smart Coater DII-29010SCTR, JEOL, Akishima, Tokyo, Japan) to prevent the PTFE samples from charging.

When a WBL is removed and recovered from PTFE surface via plasma treatment, the PTFE surface hardness increases [33]. To double-check the presence or absence of a WBL, the surface hardness was measured before and after HAP treatment using a nanoindenter (ENT-2100, Elionix, Hachioji, Tokyo, Japan). The procedure of surface hardness measurement was the same as previously reported [33]. Load–depth curves were recorded at 20-ms intervals until the maximum load was 40 μN. The surface hardness was calculated at more than 50 different points for each HAP-treated PTFE sample, then the data was displayed as histograms, as shown in Figure S5. The average surface hardness was defined as a geometric mean value in the present study, and the error bar was shown as a standard error.

#### 2.3.6. Optical Emission Spectroscopy (OES) Measurements

To compare the generation of various radicals and their ratios in He and Ar plasma treatments, OES was performed using a multichannel spectrometer (HR-4000CG-UV-NIR, Ocean Optics, Largo, FL, USA) and a light fiber (P400-2-VIS/NIR, Ocean Optics, Largo, FL, USA). The range of OES measurement was 200–1000 nm, and the exposure time of the OES measurement was 2 s.

## 3. Results and Discussion

### 3.1. Adhesion Properties

**Figure 2:**
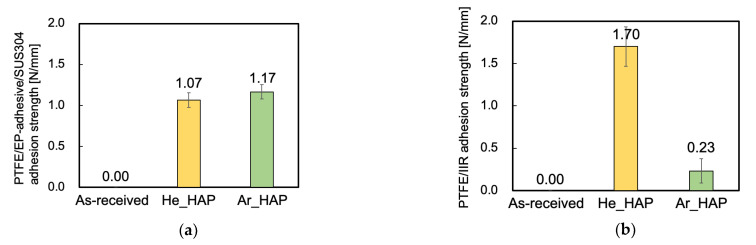
Adhesion properties of the PTFE samples HAP-treated using He or Ar gas. (**a**) PTFE/EP-adhesive/SUS304 adhesion strength and (**b**) PTFE/IIR adhesion strength.

In general, adhesive-free adhesion is more difficult than adhesive bonding. Therefore, two types of adhesion strength tests were performed on the PTFE samples: HAP-treated using He or Ar gas to compare adhesive-bonding and adhesive-free adhesion properties. Figure 2a shows the PTFE/EP-adhesive/SUS304 adhesion strengths of the PTFE samples that were HAP-treated using He or Ar gas. The as-received PTFE sample did not adhere to SUS304 with the EP adhesive at all. In contrast, both the PTFE samples HAP-treated using He and Ar strongly adhered to SUS304 with the EP adhesive. As compared to 0.3 N/mm of adhesion strength between the typical Scotch tape and others such as a SUS304 plate, over 1.0 N/mm of adhesion strength indicated significantly strong adhesion strength despite the inert nature of PTFE. These results indicated that the PTFE samples HAP-treated using He or Ar gas had a strong adhesive-bonding property and that the PTFE/EP-adhesive/SUS304 adhesion strength of the PTFE sample HAP-treated using Ar gas did not differ from that of the PTFE sample HAP-treated using He gas. Figure 2b shows the PTFE/IIR adhesion strengths of the PTFE samples HAP-treated using He or Ar gas. The as-received PTFE sample also did not adhere to IIR at all. In contrast, the PTFE samples HAP-treated using He or Ar directly adhered to IIR, which indicated that the PTFE samples HAP-treated using He or Ar gas had an adhesive-free adhesion property. However, the PTFE/IIR adhesion strength of the PTFE sample HAP-treated using Ar gas was much lower than that of the PTFE sample HAP-treated using He gas.

### 3.2. Peroxy Radical Density Ratio Calculated from ESR Spectra

**Figure 3:**
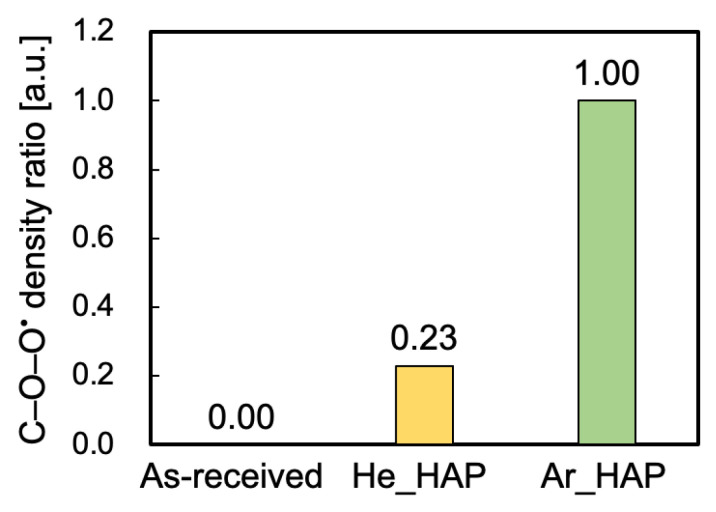
Peroxy radical density ratios calculated from the ESR spectra of the PTFE samples HAP-treated using He or Ar gas. Each radical density ratio was normalized based on the ESR spectrum of the PTFE samples HAP-treated using Ar gas.

Peroxy radical density ratios were evaluated via ESR measurements to clarify the reason for the difference in adhesion properties of He- and Ar-HAP-treated PTFE samples. In general, the larger the number of peroxy radicals is, the higher is the adhesion property of PTFE. Therefore, the number of peroxy radicals for the He-HAP-treated PTFE was expected to be larger than that for the Ar-HAP-treated PTFE because He-HAP-treated PTFE had a higher adhesive-free adhesion property than Ar-HAP-treated PTFE. Figure 3 shows the peroxy radical density ratios calculated from the ESR spectra of the PTFE samples HAP-treated using He or Ar gas. Interestingly, the peroxy radical density ratios of the Ar-HAP-treated PTFE were more than four times as high as that of the He-HAP-treated PTFE. Therefore, ESR results alone do not explain the reason for the difference in adhesion properties.

### 3.3. Surface Chemical Composition Evaluated via XPS

**Figure 4:**
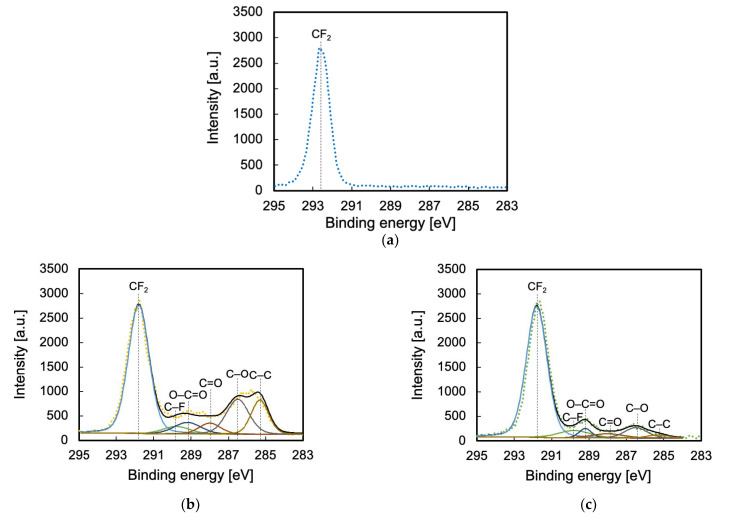
C1s-XPS spectra of the PTFE samples HAP-treated using He or Ar gas. (**a**) As-received PTFE, (**b**) He-HAP-treated PTFE, and (**c**) Ar-HAP-treated PTFE.

To clarify the reason for the difference in the adhesion properties of He- and Ar-HAP-treated PTFE, changes in surface chemical composition were evaluated via XPS measurements. In general, the higher the ratio of oxygen-containing functional groups is, the higher is the adhesion property of PTFE. Table 1 shows the atomic ratios of the PTFE samples HAP-treated using He or Ar gas. The F ratio decreased and the C and O ratios increased via He- or Ar-HAP treatment, which indicates that C–F bond scissions occurred and carbon radicals were generated, and then the carbon radicals reacted with oxygen or carbon radicals. Figure 4 shows the C1s-XPS spectra of the PTFE samples HAP-treated using He or Ar gas with heating. The O1s-XPS spectra of the PTFE samples HAP-treated using He or Ar gas with heating are shown in Figure S3. The functional-group ratios are shown in Table 2. As shown in Figure 4a, only the peak indexed to CF_2_ at 292.5 eV was detected for the as-received PTFE. For the He-HAP-treated PTFE sample, the peaks indexed to C=O–O at 289.2 eV, C=O at 288.0 eV, C–O at 286.5 eV, and C–C at 285.3 eV (Figure 4b) indicated the generation of oxygen-containing functional groups and C–C crosslinks. In contrast, for the Ar-HAP-treated PTFE sample, the sum of oxygen-containing functional group ratio (14%) and C–C ratio (2%) significantly decreased (Figure 4c) as compared to those ratios of the He-HAP-treated PTFE sample (28% and 12%), indicating that oxygen-containing functional groups and C–C crosslinks were generated upon Ar plasma treatment, but the ratios of oxygen-containing functional groups and C–C crosslinks were not so high. The ratios of oxygen-containing functional groups and C–C crosslinks contributed to the difference in the adhesive-free adhesion properties of He- and Ar-HAP-treated PTFEs.

### 3.4. Surface Modification Depth Evaluated via GCIB–XPS

**Figure 5:**
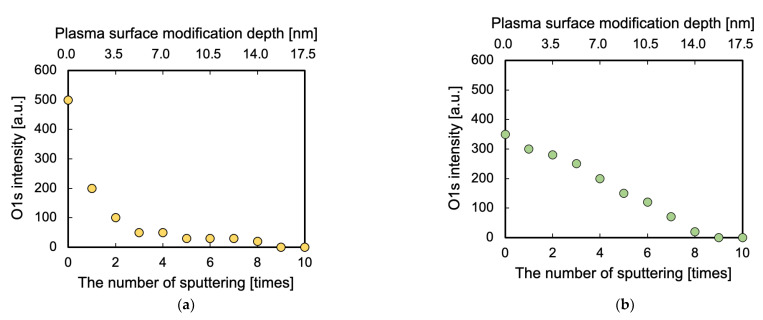
Change in the O1s-XPS peak intensity with increasing the number of sputtering for the PTFE samples HAP-treated using He or Ar gas. (**a**) He-HAP-treated PTFE and (**b**) Ar-HAP-treated PTFE. The upper X-axis showing plasma surface modification depth was calculated by multiplying the GCIB sputtering rate (1.75 nm/time = 3.5 nm/min) and the number of sputtering.

To investigate the relation between the surface modification depth and adhesion properties of PTFE, surface modification depth was measured via GCIB–XPS measurement. Figure 5 shows the change in the intensity of the O1s-XPS peak with increasing in the number of sputtering for the PTFE samples HAP-treated using He or Ar gas. Both the intensities of the O1s-XPS peaks decreased by increasing the number of sputtering; then, they reached zero at the number of sputtering of 9 times, indicating that the surface modification layer generated by He- or Ar-HAP treatment was etched and removed completely from the PTFE sample at the number of sputtering of 9 times. The number of sputtering was converted into the surface modification depth, which was 16 ± 2 nm for He- or Ar-HAP-treated PTFE samples, respectively. Although the initial O1s-XPS intensity and the decreasing rate of the O1s-XPS intensity differed between He and Ar plasma-treated PTFE samples, their surface modification depths did not substantially differ, indicating that the cause of the difference in the adhesive-free adhesion properties of He- and Ar-HAP-treated PTFE is not the surface modification depth.

### 3.5. Confirmation of WBL Removal by SEM Observation and Surface Hardness

**Figure 6:**
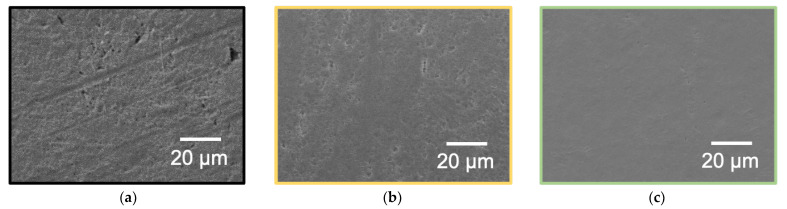
SEM images of the PTFE samples HAP-treated using He or Ar gas. (**a**) As-received PTFE, (**b**) He HAP-treated PTFE, and (**c**) Ar HAP-treated PTFE.

SEM observations were performed at three different points at least, and the resemble tendency was confirmed. Therefore, the representative SEM images of the PTFE sample HAP-treated using He or Ar gas are shown in Figure 6. For the as-received PTFE, many cutting scratches indexed to a WBL are observed in Figure 6a, as previously reported [24]. For both the He- and Ar-HAP-treated PTFE samples, no cutting scratches are observed in Figure 6b,c indicating that the WBL was removed and recovered from both HAP-treated PTFE surfaces. Although the time of the He-HAP treatment was the same as that of the Ar-HAP treatment, the number of pits on the Ar-HAP-treated PTFE seemed to be less than that on the He-HAP-treated PTFE, suggesting that the remaining WBL on the Ar-HAP-treated PTFE surface did not contribute to the lower adhesion property of the Ar-HAP-treated PTFE than that of He-HAP-treated PTFE.

**Figure 7:**
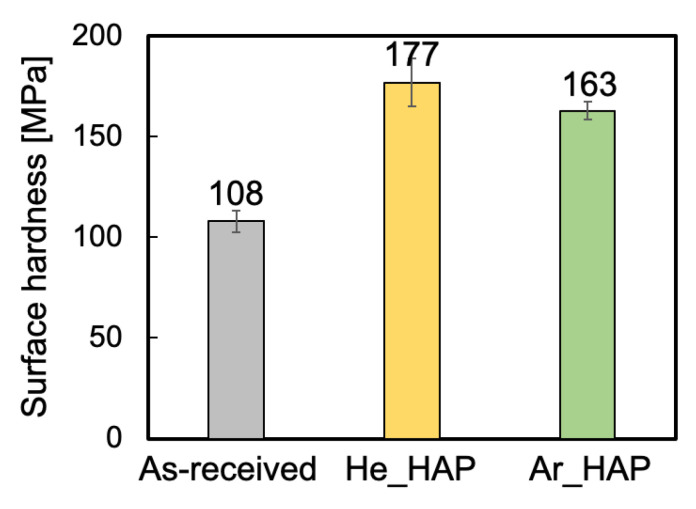
Surface hardness of the PTFE samples HAP-treated using He or Ar gas.

To double-check the WBL removal and investigate the relation between the surface hardness and adhesion properties of PTFE, surface hardness was measured using nanoindentation. Figure 7 shows the surface hardness of the PTFE sample HAP-treated using He or Ar gas. The surface hardness increased to higher than 160 MPa via He- or Ar-HAP treatment, as compared to as-received PTFE (108 MPa). The combination of the results of the SEM observation and the surface hardness indicated that the WBL was removed from the He- or Ar-HAP-treated PTFE surface, respectively. It was reported that the surface hardness was 155 MPa when the plasma-treated PTFE surface had neither C–C crosslinks nor the WBL, while the surface hardness was 177 MPa when the plasma-treated PTFE surface had C–C crosslinks but no WBL [33]. In short, the report demonstrated that the factors that increase the surface hardness include the removal of a WBL and the increase in C–C crosslinks; the removal of a WBL induced an increase of 45 MPa, and the increase in C–C crosslinks induced an increase of 22 MPa in surface hardness. The combination of the results of the XPS spectra and the surface hardness in this study indicated that the difference in the number of C–C crosslinks induced the difference in surface hardness for He- and Ar-HAP-treated PTFE samples. Therefore, the adhesive-free adhesion property of He-HAP-treated PTFE was higher than that of Ar-HAP-treated PTFE because of the difference in surface hardnesses induced via the difference in the number of C–C crosslinks.

### 3.6. Influence of VUV/UV Irradiation on the Generation of Peroxy Radicals and Surface Chemical Composition

**Figure 8:**
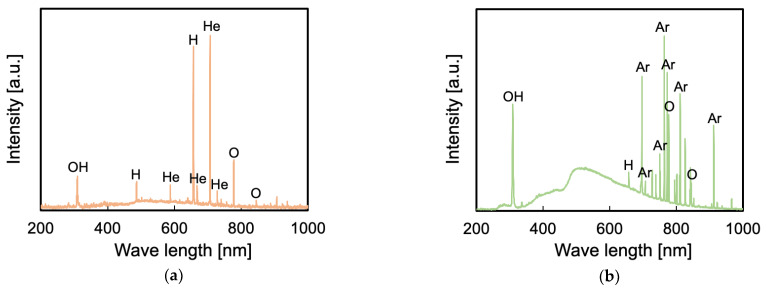
OES spectra in the plasma treatment without heating (**a**) using He gas and (**b**) using Ar gas. Each measurement period was 2 s.

To investigate the influence of VUV/UV irradiation on the generation of peroxy radicals, OES and ESR spectra were obtained. Figure 8 shows the OES spectra in the plasma treatment using He or Ar gas without heating. As seen in Figure 8a, the peaks indexed to helium (He) were observed at 587, 667, 706, and 728 nm, the peaks indexed to oxygen (O) were observed at 777 and 845 nm, the peaks indexed to hydrogen (H) were observed at 486 and 656 nm, and the peak indexed to OH was observed at 309 nm. As seen in Figure 8b, the peaks indexed to argon (Ar) were observed at 697, 707, 750, 764, 773, 810, and 912 nm, the peaks indexed to oxygen (O) were observed at 777 and 845 nm, the peak indexed to hydrogen (H) was observed at 656 nm, and the peak indexed to OH was observed at 309 nm [34,35,36]. The intensity of the peak indexed to OH should be noted because shorter-wavelength light has a higher energy and is likely to cut C–F and C–C bonds not only on the surface but also in the bulk of PTFE samples. The intensity of the peak indexed to OH for Ar plasma-treated PTFE was much higher than that of the peak indexed to OH for He plasma-treated PTFE. This result shows that the Ar plasma treatment has stronger VUV/UV irradiation and induces more scissions of C–F and C–C bonds in PTFE bulk than He plasma treatment.

**Figure 9:**
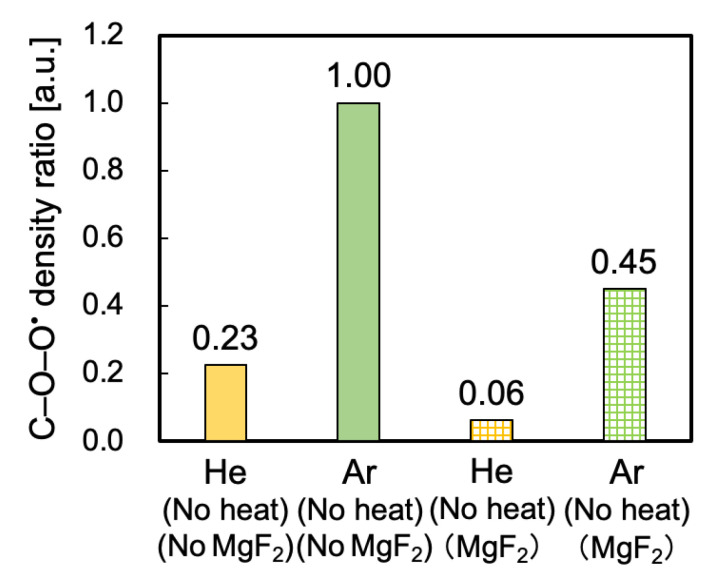
Peroxy radical density ratios calculated from the ESR spectra of the PTFE samples plasma-treated using He or Ar gas without or with an MgF_2_ substrate and without using a heater. Each radical density ratio was normalized based on the ESR spectrum of the PTFE samples plasma-treated using Ar gas without an MgF_2_ substrate.

Figure 9 shows the peroxy radical density ratios calculated from the ESR spectra of the PTFE samples plasma-treated using He or Ar gas without or with the MgF_2_ substrate and without using a heater. The peroxy radical density ratio of the Ar plasma-treated PTFE was much higher than that of the He plasma-treated PTFE, indicating that the influence of the VUV/UV irradiation for the Ar plasma-treated PTFE was higher than that of the He plasma-treated PTFE.

**Figure 10:**
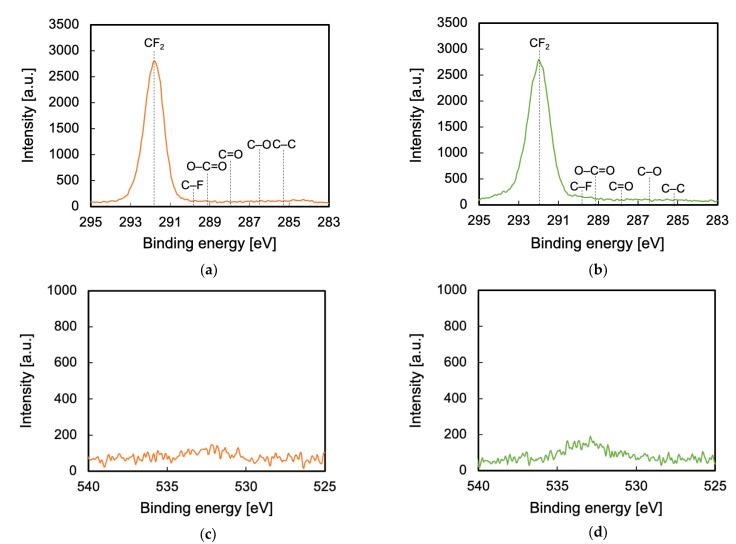
XPS spectra of the plasma-treated PTFE samples covered with an MgF_2_ substrate and without using a heater. (**a**) C1s-XPS of the He plasma-treated PTFE, (**b**) C1s-XPS of the Ar plasma-treated PTFE, (**c**) O1s-XPS of the He plasma-treated PTFE, and (**d**) O1s-XPS of the Ar plasma-treated PTFE.

To investigate the influence of VUV/UV irradiation on surface chemical composition, XPS measurements were also performed. Figure 10a,b show the C1s-XPS spectra of the PTFE samples plasma-treated using He or Ar gas with the MgF_2_ substrate and without using a heater. Both the C1s-XPS spectra had only the peak indexed to CF_2_ at 291.8 eV, and the shape of the spectra resembled that of the C1s-XPS spectrum for the as-received PTFE in Figure 4a. Figure 10c,d show the O1s-XPS spectra of the PTFE samples plasma-treated using He or Ar gas with the MgF_2_ substrate and without using a heater. Both the O1s-XPS spectra had a weak and broad peak indexed to O–C and O=C at 535–530 eV. These results indicated that the influence of VUV/UV irradiation on the surface chemical composition was weak.

### 3.7. Difference between He- and Ar-HAP Treatments

**Figure 11:**
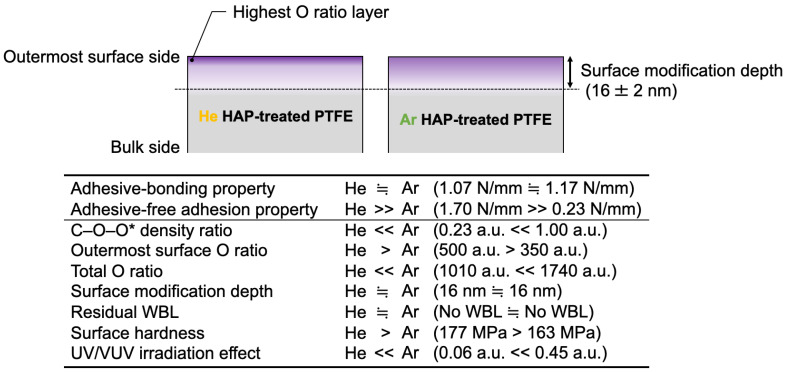
Schematic diagram of He- and Ar-HAP-treated PTFE surfaces and the summarized results.

The difference between He- and Ar-HAP treatments is summarized in Figure 11. The adhesive-bonding properties were similar, but the adhesive-free adhesion properties were very different. The difference of the adhesive-free adhesion properties was due to the higher outermost surface O ratio containing peroxy radicals and oxygen-containing functional groups and higher surface hardness via C–C crosslinks, but not due to the number of peroxy radicals, total O ratio, or surface modification depth. These findings would be good guideline for improving an adhesive-free adhesion property of not only fluoropolymers but also other polymers. Especially, the data on the relation between the adhesion property and surface modification depth would be useful because there are few reports on the relation. In addition, the difference in gas species in HAP treatment induced the difference in UV/VUV irradiation effects. This behavior of PTFE toward UV/VUV irradiation in HAP treatment explained the contradiction between the adhesive-free adhesion properties and peroxy radical density ratios.

## 4. Conclusions

The influences of gas species in HAP treatment on the adhesion property of PTFE were investigated. In adhesive bonding, the adhesion strength of Ar-HAP-treated PTFE/EP-adhesive/SUS304 was almost the same as that of He-HAP-treated PTFE/EP-adhesive/SUS304, indicating that the adhesive-bonding property of Ar-HAP-treated PTFE was the same as that of He-HAP-treated PTFE. Moreover, in adhesive-free adhesion, the adhesion strength of Ar-HAP-treated PTFE/IIR was much lower than that of He-HAP-treated PTFE/IIR. Thus, the replacement from He to Ar gas in HAP treatment is possible in adhesive bonding, but the replacement is impossible in adhesive-free adhesion. The reason for the difference in the adhesion properties of Ar and He-HAP-treated PTFE samples was also investigated. The two treatment methods led to differences in the peroxy radical density ratio, surface chemical composition, surface hardness, and surface morphology of PTFE. The surface chemical composition, especially high ratios of oxygen-containing functional groups and C–C crosslinks, significantly contributed to the difference in the adhesive-free adhesion properties. Although the peroxy radical density ratio of the Ar-HAP-treated PTFE was higher than that of the He-HAP-treated PTFE, the adhesive-free adhesion property of the Ar-HAP-treated PTFE was lower than that of the He-HAP-treated PTFE. The contradiction was explained by the VUV/UV irradiation during plasma treatment and demonstrated by the plasma treatment using an MgF_2_ substrate. Although Ar-HAP treatment was not effective to improve the adhesive-free adhesion of PTFE, it improved the adhesive-bonding property of PTFE. These results suggest that Ar-HAP treatment should be used in the case of adhesive bonding, whereas He-HAP treatment should be used in the case of adhesive-free adhesion. Except for medical and food fields, an adhesive-bonding is often used in various applications. Replacement of a process gas from He to Ar gas in HAP treatment would bring a cost reduction in the case of an adhesive-bonding between PTFE and other materials. In summary, gaseous species can be used accordingly for targeted applications. Next, we will further develop an Ar-HAP treatment to improve the adhesive-free adhesion of PTFE and search for an alternative gas that can replace He to expand the applications of HAP treatment. In the present study, HAP treatment was applied to an ordinary flat PTFE sheet; in the future, HAP treatment would be applied to several forms of fluoropolymers such as porous and powder materials.

## Figures and Tables

**Table 1:** Atomic ratios of the PTFE samples HAP-treated using He or Ar gas.

Sample	C [at.%]	O [at.%]	F [at.%]
As-received	31.3	0.0	68.7
He_HAP	43.5	3.1	53.4
Ar_HAP	37.4	0.8	61.8

**Table 2:** Functional-group ratios of the PTFE samples HAP-treated using He or Ar gas.

Functional Group Type	CF_2_	C–F	O–C=O	C=O	C–O	C–C
Binding energy [eV]	291.8	289.8	289.2	288.0	286.5	285.3
As-received [%]	100	0	0	0	0	0
He-HAP [%]	55	4	6	5	17	12
Ar-HAP [%]	77	7	3	4	7	2

## Data Availability

The data presented in this study are available in the article.

## Supplementary Materials

The following are available online at https://www.mdpi.com/article/10.3390/polym13234266/s1, Figure S1: Electron spin resonance (ESR) spectra of the PTFE samples HAP-treated using He or Ar gas. (a) As-received PTFE, (b) He-HAP-treated PTFE, and (c) Ar-HAP-treated PTFE. Figure S2: Electron spin resonance (ESR) spectra of the PTFE samples plasma-treated using He or Ar gas without or with the MgF_2_ substrate and without using a heater. (a) He plasma-treated PTFE without an MgF_2_ substrate, (b) Ar plasma-treated PTFE without an MgF_2_ substrate, (c) He plasma-treated PTFE with an MgF_2_ substrate, and (d) Ar plasma-treated PTFE with an MgF_2_ substrate. Figure S3: O1s-XPS spectra of the PTFE samples HAP-treated using He or Ar gas. (a) As-received PTFE, (b) He-HAP-treated PTFE, and (c) Ar-HAP-treated PTFE. Figure S4. Gas cluster ion beam (GCIB) sputtering depth observed using a scanning white-light interferometer (SWLI). (a) SWLI image of the front view and (b) cross-sectional view around the GCIB sputtering hall. Figure S5. Surface hardness histograms of the PTFE samples plasma-treated using He or Ar gas with heating. (a) As-received PTFE, (b) He heat-assisted-plasma (HAP)-treated PTFE, and (c) Ar-HAP-treated PTFE.
