# Supplementary material for: Effects of He and Ar Heat-Assisted Plasma Treatments on the Adhesion Properties of Polytetrafluoroethylene (PTFE)
Source: Polymers (Basel). 2021 Dec 6;13(23):4266. doi: 10.3390/polym13234266 (PMC8659427; doi:10.3390/polym13234266)
Supplement: Supplementary file 1 [file polymers-13-04266-s001.zip › polymers-1421622-supplementary.pdf]

polymers

Supplementary Materials: Effects of He and Ar Heat-Assisted Plasma Treatments on the Adhesion Properties of Polytetrafluoroethylene (PTFE)

Yuji Ohkubo, Yuki Okazaki, Masafumi Shibahara, Misa Nishino, Yosuke Seto, Katsuyoshi Endo, and Kazuya Yamamura

Contents

- ESR spectra before and after HAP treatment ······ Figure S1
- ESR spectra without using a heater ······ Figure S2
- O1s-XPS spectra before and after HAP treatment ······ Figure S3
- GCIB sputtering depth ······ Figure S4
- Surface hardness histograms ······ Figure S5

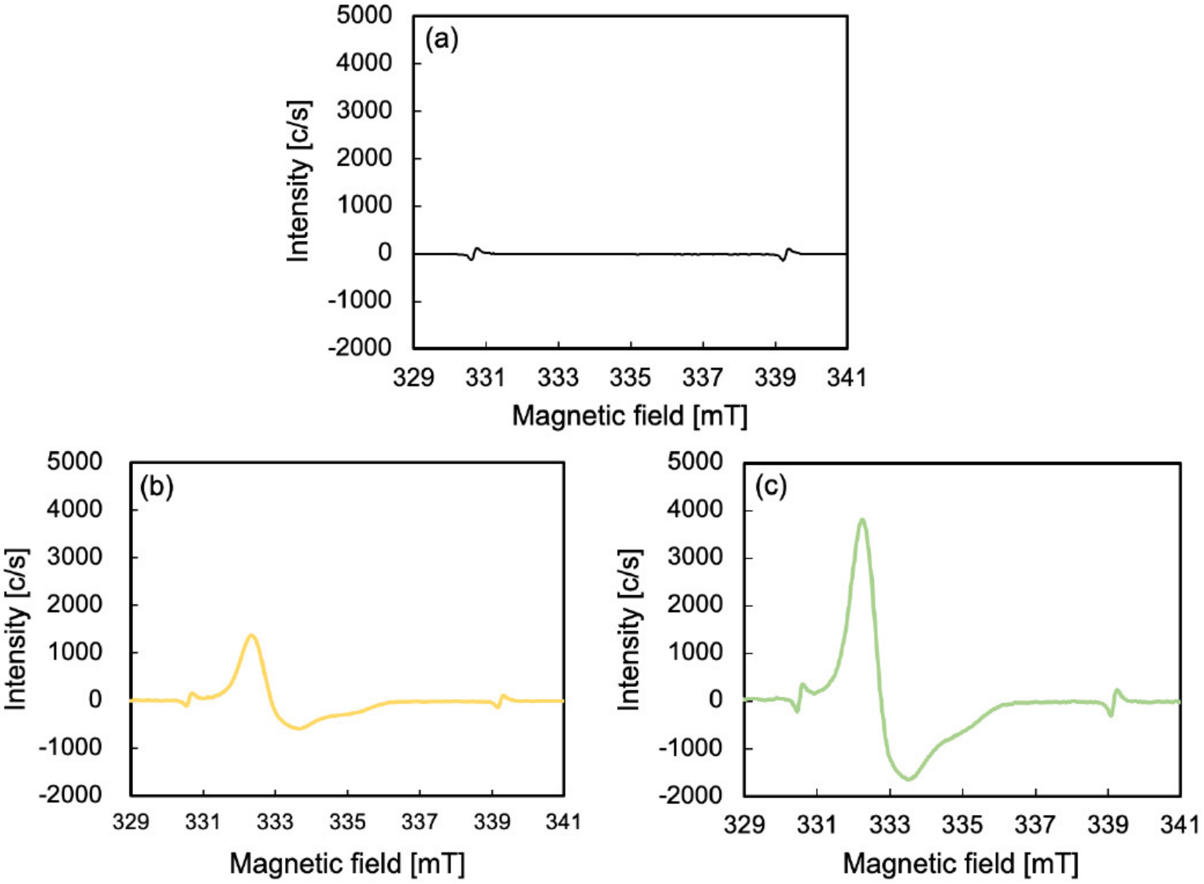

Figure S1. Electron spin resonance (ESR) spectra of the PTFE samples HAP-treated using He or Ar gas. (**a**) As-received PTFE, (**b**) He HAP-treated PTFE, and (**c**) Ar HAP-treated PTFE.

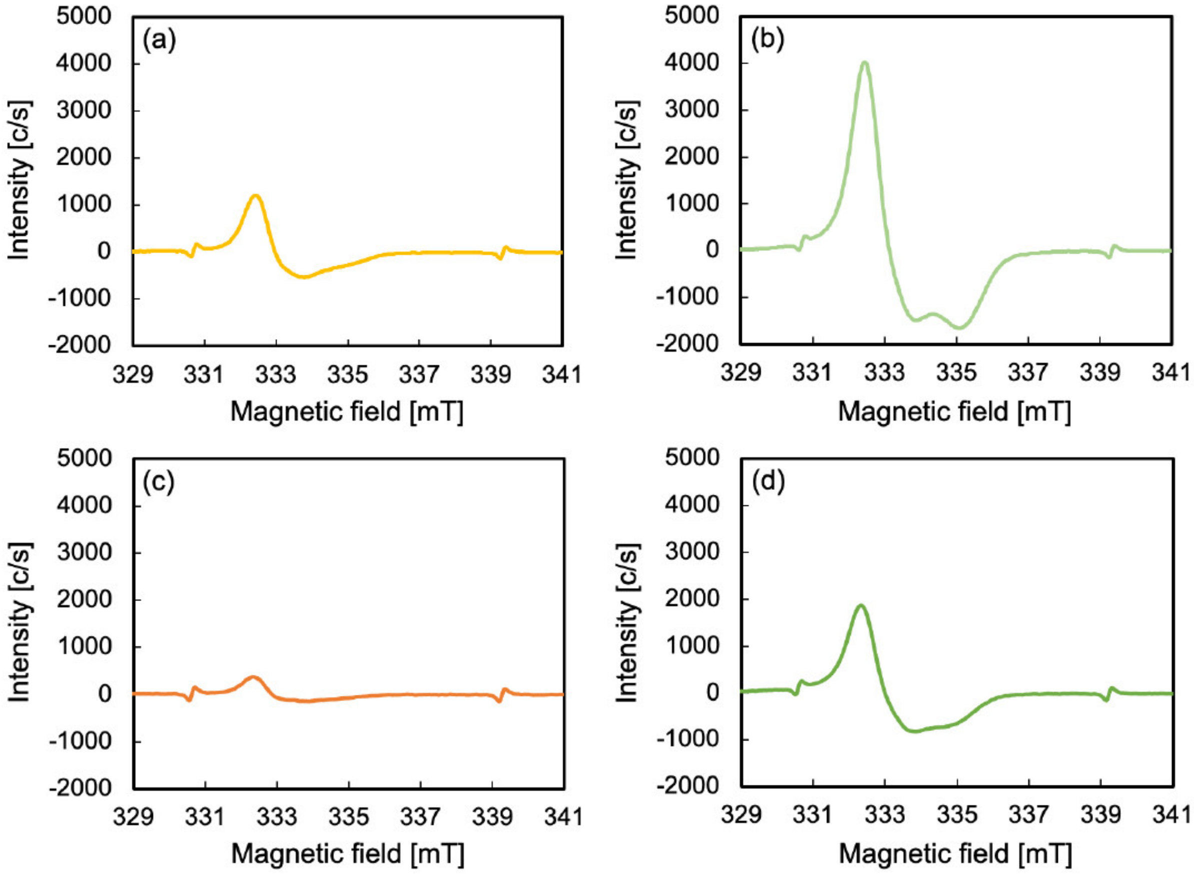

Figure 2. Electron spin resonance (ESR) spectra of the PTFE samples plasma-treated using He or Ar gas without or with the MgF_2 substrate and without using a heater. (**a**) He plasma-treated PTFE without an MgF_2 substrate, (**b**) Ar plasma-treated PTFE without an MgF_2 substrate, (**c**) He plas-ma-treated PTFE with an MgF_2 substrate, and (**d**) Ar plasma-treated PTFE with an MgF_2 substrate.

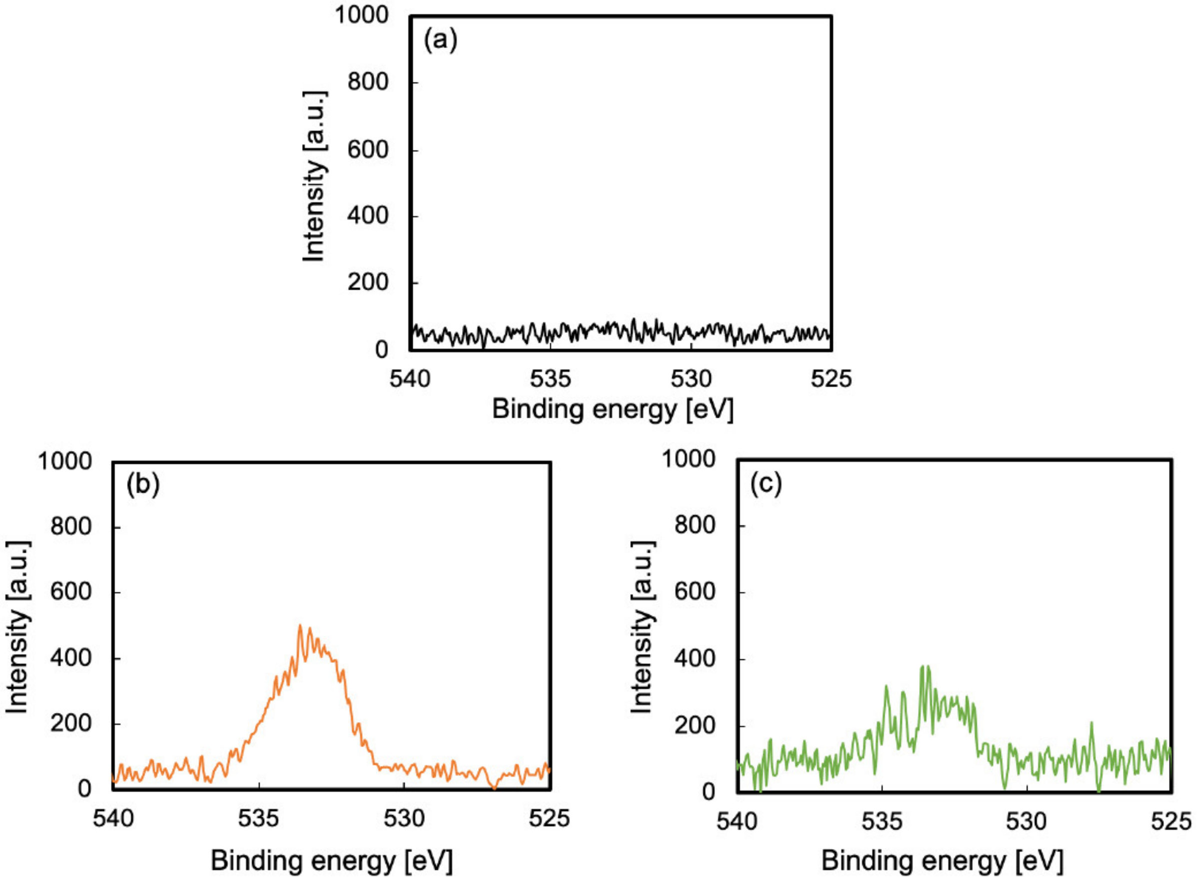

Figure 3. O1s-XPS spectra of the PTFE samples HAP-treated using He or Ar gas. (**a**) As-received PTFE, (**b**) He HAP-treated PTFE, and (c) Ar HAP-treated PTFE.

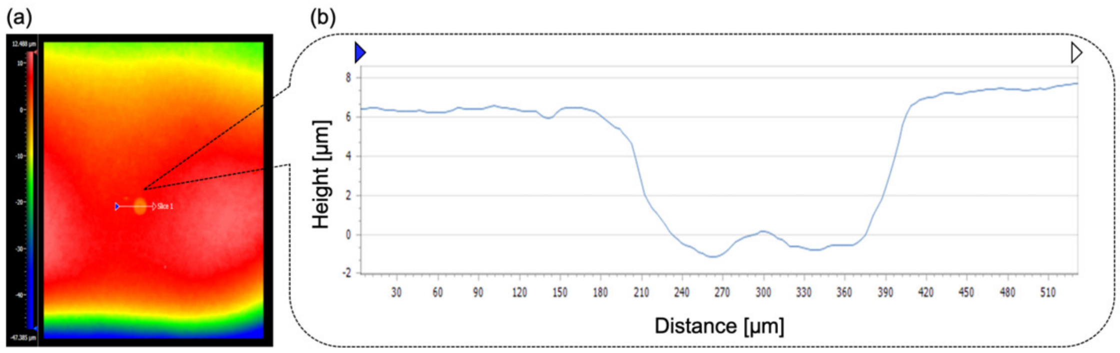

Figure S4. Gas cluster ion beam (GCIB) sputtering depth observed using a scanning white-light interferometer (SWLI) (a) SWLI image of the front view and (b) cross-sectional view around the GCIB sputtering hall.

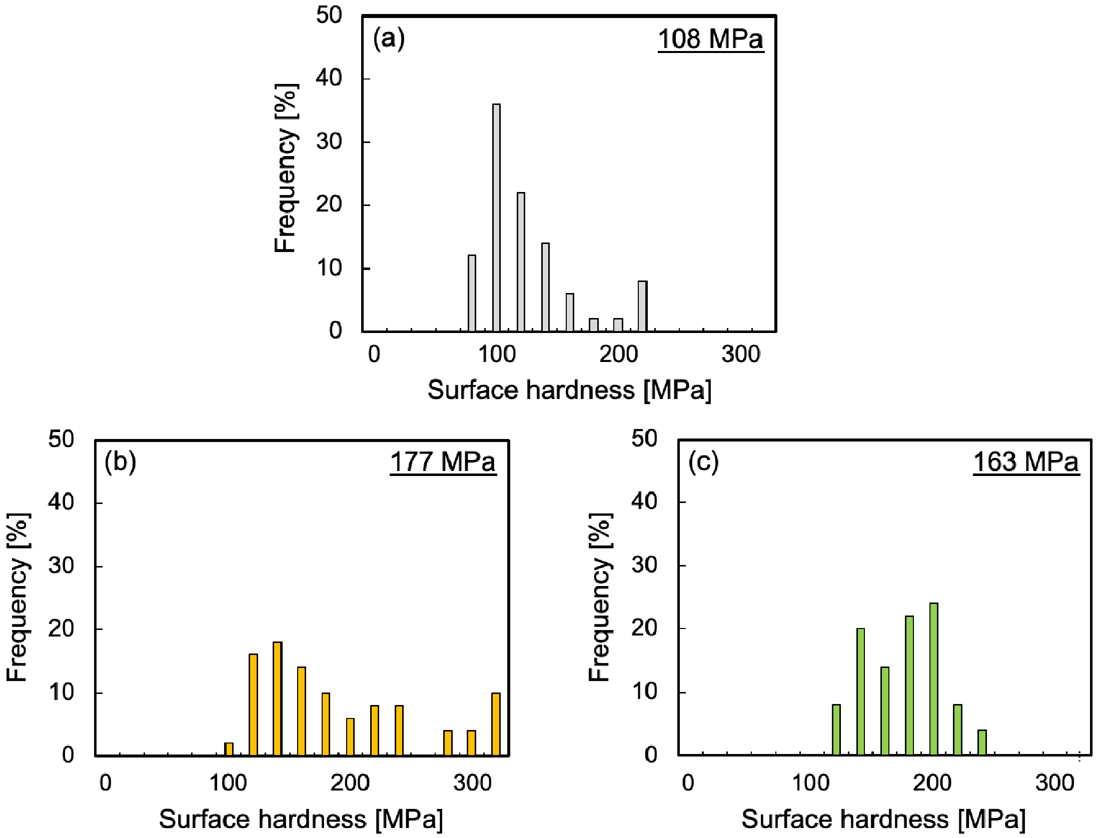

Figure S5. Surface hardness histograms of the PTFE samples plasma-treated using He or Ar gas with heating. (**a**) As-received PTFE, (**b**) He heat-assisted-plasma (HAP)-treated PTFE, and (c) Ar HAP-treated PTFE.
